# Current advances of epigenetics in periodontology from ENCODE project: a review and future perspectives

**DOI:** 10.1186/s13148-021-01074-w

**Published:** 2021-04-26

**Authors:** Young-Dan Cho, Woo-Jin Kim, Hyun-Mo Ryoo, Hong-Gee Kim, Kyoung-Hwa Kim, Young Ku, Yang-Jo Seol

**Affiliations:** 1 Department of Periodontology, School of Dentistry and Dental Research Institute, Seoul National University and Seoul National University Dental Hospital, Yeongeon-dong, Jongno-gu, Seoul, 03080 Korea; 2 Department of Molecular Genetics, School of Dentistry and Dental Research Institute, Seoul National University, Seoul, Korea; 3 Biomedical Knowledge Engineering Laboratory, School of Dentistry and Dental Research Institute, Seoul National University, Seoul, Korea

**Keywords:** Chronic disease, Epigenetics, ENCODE project, Next-generation sequencing, Periodontology, Personalized medicine

## Abstract

**Background:**

The Encyclopedia of DNA Elements (ENCODE) project has advanced our knowledge of the functional elements in the genome and epigenome. The aim of this article was to provide the comprehension about current research trends from ENCODE project and establish the link between epigenetics and periodontal diseases based on epigenome studies and seek the future direction.

**Main body:**

Global epigenome research projects have emphasized the importance of epigenetic research for understanding human health and disease, and current international consortia show an improved interest in the importance of oral health with systemic health. The epigenetic studies in dental field have been mainly conducted in periodontology and have focused on DNA methylation analysis. Advances in sequencing technology have broadened the target for epigenetic studies from specific genes to genome-wide analyses.

**Conclusions:**

In line with global research trends, further extended and advanced epigenetic studies would provide crucial information for the realization of comprehensive dental medicine and expand the scope of ongoing large-scale research projects.

## Background

The Encyclopedia of DNA Elements (ENCODE) project has advanced our knowledge of the functional elements in the genome and epigenome, including chromatin organization and gene regulatory elements [1]. Along with this trend of research, epigenetic approaches to dental research have been activated, suggesting a bright future direction for an in-depth understanding of the diseases. Following the ENCODE project, global epigenome research projects, such as the NIH Roadmap Epigenome Program and International Human Epigenome Consortium, emphasized the importance of epigenetic research for understanding human health and disease [2]. Current international consortia show an improved interest in the importance of oral health with systemic health, and the trend of epigenetic research continues in the dental field. Among them, a number of epigenetic studies have been conducted in the field of periodontology [3, 4]. Periodontal disease, a representative chronic oral disease common in the elderly population characterized by inflammation and periodontal tissue destruction, is mainly caused by a multifactorial bacterial infection [5]. Periodontal diseases are also related to systemic chronic diseases, such as non-communicable diseases, cardiovascular disease, diabetes mellitus, and other metabolic syndromes [6–8]. These chronic diseases are usually persistent or long-lasting and are related to various environmental conditions, suggesting the possibility of association with epigenetic changes [9]. Therefore, further extended and advanced epigenetic studies in periodontology would provide crucial information for the realization of comprehensive dental medicine and expand the scope of ongoing large-scale research projects. In this review, we describe the contributions of the ENCODE project and cutting-edge technologies to human health, broadly, and periodontology, in particular. Furthermore, we establish the link between epigenomic changes and periodontal diseases based on recent researches and seek the future direction of development.

## Periodontal disease: development and progression

**Fig. 1:**
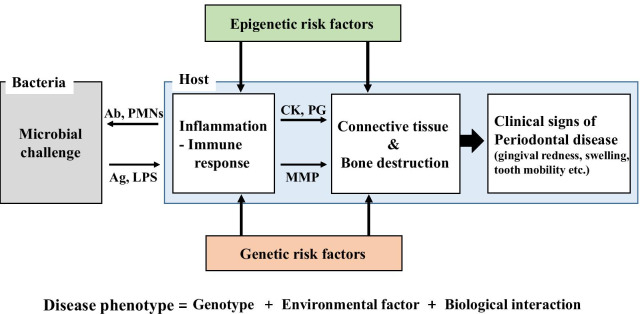
Periodontal disease: development and progression. Generally, periodontal disease is initiated by microbial challenges and progresses with an immuno-inflammatory response, which deteriorates connective tissue and causes bone destruction. During this process, genetic risk factors work in conjunction with epigenetic factors, and disease phenotype appears as comprehensive product of genotypic, environmental, and biological interaction. Ab, antibody; PMNs, polymorphonuclear cells; Ag, antigen; LPS, lipopolysaccharides; CK, cytokine; PG, prostaglandin; MMP, matrix metalloproteinases

Periodontitis is a representative, multifactorial, and chronic inflammatory disease with genetic and epigenetic factors (Fig. 1). It is well known that oral bacteria in dental plaque induce periodontal disease and the consequent progression is associated with many risk factors, such as genetics, lifestyle, and systemic disease. If periodontitis is not treated, several clinical signs, such as alveolar bone destruction, periodontal attachment loss, and tooth mobility, would follow [10].

### Microbial challenge

Periodontal diseases are closely related to a wide ranges of microbiome profiles depending on the type of disease [11]. The microorganisms in the oral cavity have been named as oral microbiota, oral microflora, and oral microbiome recently. Oral microbiome refers to the collective genetic materials of oral microorganisms. The term microbiome is defined as the ecological community of microorganism residents and is widely used by the Human Microbiome Project [12, 13]. Socransky and Haffajee [14] classified the pathogens as five complexes: yellow, green, purple, orange, and red, according to their presence in the biofilm. Generally, bacteria of yellow, green, purple complexes indicate comparatively healthy gingival tissue, whereas red and orange complexes indicate periodontal pathogens [15]. Periodontal tissue destruction is predominantly caused by the host inflammatory reaction to the microbial challenge (Fig. 1). In the oral microbial biofilm, over 500 species of bacteria are present, forming a dynamic community which adheres to teeth and periodontal tissue, and progresses into microbial colonization [16]. Therefore, a fundamental treatment of periodontal disease such as scaling or root planing is to remove dental plaque and calculus and reduce bacterial invasion of periodontal tissue.

### Host response to microbial challenge

In addition to the microbial community in the environmental flux, pathogen–host interaction is crucial to control periodontal disease. The oral epithelium is the first host cell to contact bacteria and acts as a physical and chemical barrier to separate the body from the microbial infection [17]. The gingiva is divided into two main parts; epithelial and connective tissue, which protects the surrounding of teeth and alveolar bone. Generally, junctional epithelium consists of specialized structures, such as adherence and gap junctions, which provide a favorable environment to the host immune system. Epithelial cells and leukocytes stimulate their antimicrobial mechanism producing antibacterial peptides, such as α- and β-defensins, and cathelicidin, in response to an attack by pathogens [18]. Furthermore, various cytokines including interleukin-1,6,8 and tumor necrosis factor-α are involved in the development of an inflammatory response [19]. Therefore, host immune defense factors are essential in determining disease susceptibility, which is closely related to variation of signaling pathways and pathogen recognition receptors, and dictates the host response to the same bacterial infection. Thus far, genetics has been limited and unable to explain this difference; hence, epigenetics was introduced to clarify the distinction of host response. Genetic factors are undoubtedly very important in the development of disease, but genetic changes can only be a risk factor when challenged by exogenous substances. Without inducing any changes in DNA sequences, epigenetic modifications can alter gene expression patterns which elicit a diverse host response [20]. Therefore, studies have been focused on regulation of gene expression, immune response, and disease susceptibility to the infections by epigenetic factors [21]. In addition, various efforts were made to find new epigenetic biomarkers for the diagnosis, prognosis, and treatment [22].

## Changing research trend from genetics to epigenetics in periodontology

With advancing age, most people suffer from periodontal disease, e.g., chronic periodontitis, and one or more other chronic systemic diseases could be involved [23]. Periodontitis is a representative chronic disease that is usually initiated by a bacterial challenge to the host (Fig. 1). Clinical signs of susceptibility and recurrence after treatment tend to be related to the causal microorganisms and inflammation [24]. Extensive studies have focused on genetic factors involved in periodontal disease, e.g., genes affecting inflammatory and immune responses, to develop treatment modalities [25]. As the immune system plays a potent role in the pathogenesis of periodontitis, many studies have identified genetic polymorphisms associated with immunity [26]. Genetic factors are mainly associated with periodontal disease-related syndromes such as Papillon-Lefevre and Chediak-Higashi [27]. The genetic basis of periodontitis has been supported by familial aggregation or twin studies [28] and candidate gene studies [29]. Furthermore, genome-wide association (GWAS) studies have identified that genetic polymorphisms are associated with disease-related phenotypes [30–32]. GWAS studies have identified the genes for susceptibility of periodontal disease; NCR2 and EMR1 in chronic periodontitis [33] and GLT6D1 in aggressive periodontitis [34]. However, it has not been fully understood how genes affect pathogenesis. Many disease characteristics have a genetic basis, but variation in gene expression with respect to environmental risk factors is not fully understood [35]. Similar to other chronic systemic diseases, periodontitis is typically associated with both genetic and epigenetic factors, including effects of environmental factors [22, 36]. Studies have shown that the pathogenesis of periodontal disease is complex, and susceptibility to periodontitis varies substantially among individuals with the same microbial infection [37]. Additionally, the host response varies according to environmental conditions. Among many environmental factors, smoking is the best known modifiable risk factor for periodontal disease [37]. A strong association between smoking and periodontal disease has been reported [38, 39]. The association between periodontal condition and other environmental factors, such as dietary habits, long-term medication, and systemic disease, has been discussed; however, precise molecular mechanism has not been clearly established [40]. Unlike genetic information, which is static within an individual, epigenetic information can be actively changed by environmental conditions. Accordingly, epigenetics might be a crucial tool to explain how the environment influences the host response via changes in gene expression. As epigenetic research approach in the dental field is emerging, further studies of inflammation and disease biomarkers as well as studies of the effects of environmental factors on periodontal disease would bring the field into a new era [41, 42].

## Epigenetics: linking environmental niches to human diseases

Many scientists were hoping that unresolved biological questions about genetic diseases would be settled after the completion of the Human Genome Project (HGP); however, these data did not provide clear answers [43, 44]. Genomic sequences alone could not explain how DNA acts within chromatin and chromosomes, even though it is critical for cellular differentiation and development. Therefore, new powerful methods to clarify the relationships of biological phenomena with genomic information were needed. Over time, a number of biological phenomena have been linked to epigenetics [45]. To understand phenotypes and biological processes for which genetic explanations are insufficient, epigenetics has been quickly growing, with an understanding that environment factors can interact with the genome, resulting in epigenetic changes [46].

**Fig. 2:**
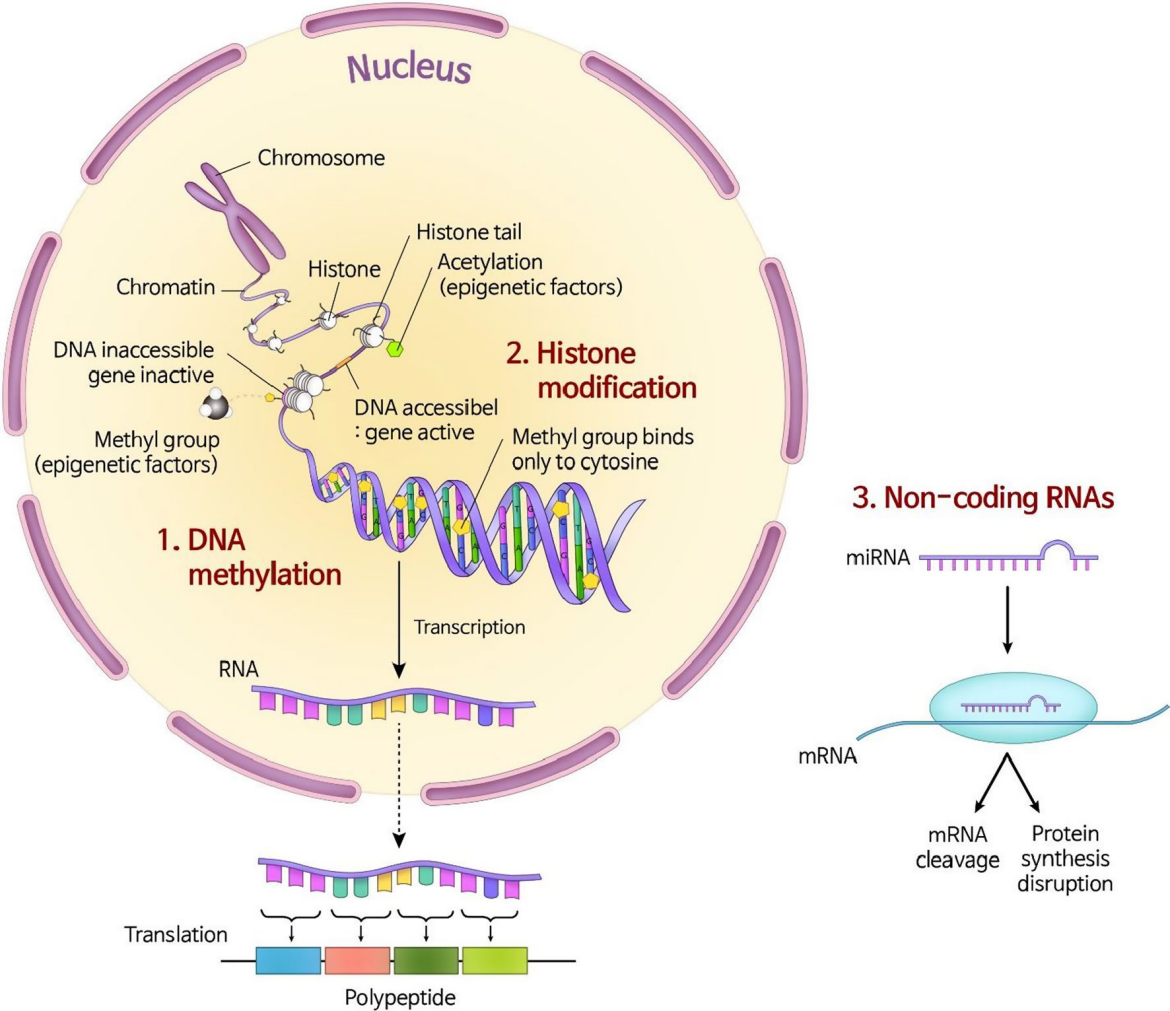
Mechanisms of epigenetics. The epigenetic modifications cause alterations in gene expression without directly altering the DNA sequence. In general, DNA methylation causes a segment of DNA to associate more closely with a histone complex. This prevents transcription factors from binding to a DNA sequence, such as a promoter, resulting in reduced expression of a specific gene. In contrast, histone acetylation weakens the interaction between the histone complex and DNA, allowing transcription factors to bind to the promoter and increase gene expression. Non-coding RNAs also play a crucial role in the regulation of gene expression

Epigenetics is the study of phenotypic changes caused by alterations in gene expression, without changes in the DNA sequence [45]. Epigenetic changes mainly occur as an array of molecular mechanisms affecting both chromatin and DNA (Fig. 2). Three representative mechanisms, DNA methylation, histone modifications, and non-coding RNA-associated gene silencing, clearly induce and maintain epigenetic changes [45, 47, 48]. These epigenetic changes could occur naturally or could be influenced by environmental factors, individual lifestyle, and chemical and physical factors. The changes could be localized to one generation and transmitted across generations. Increasing evidence based on animal and human studies indicates that prenatal and early postnatal environmental factors affect the risk of chronic diseases and behavioral disorders in adults [21, 49–52]. New and ongoing studies are continuously investigating the role of epigenetics in a variety of human diseases.

## Encyclopedia of DNA Elements (ENCODE) project

To provide insight beyond the HGP, the ‘ENCODE’ project has recently been launched after the completion of the HGP in 2003 by a worldwide collaborative research group funded by the National Human Genome Research Institute [1, 53, 54]. The aim of ENCODE is to generate a comprehensive list of all functional elements that act at the protein and RNA levels, including genes, transcripts, and transcriptional regulatory regions, together with their chromatin histone states and DNA methylation patterns in the human genome [55]. In other words, while the HGP sequenced the DNA in the human genome, the aim of ENCODE is to interpret these sequence data. Because about 20,000 genes provide the information to make proteins, accounting for only about 1% of the human genome, ENCODE was introduced to understand the remaining 99% of the genome. Initially, more than 80% of the genome was mischaracterized as ‘junk DNA,’ but the non-coding genome actually has a crucial role in regulating gene activity and expression. The modulation of gene activity may regulate transcription, translation, and cellular functions and result in disease. Therefore, the ultimate goal of the ENCODE project is to link variation in gene expression to the development of disease [54]. At first, ENCODE pilot project in 2003 focused on a defined 1% of human genome sequence [56]; then, by 2012, ENCODE consortium expanded to the entire genome with an integrated ENCODE map allowing to comprehend the biochemical functions to 80% of the genome [57]. In 2009, a limited range of mouse ENCODE project was launched with the high value and use of experimental mice [58]. Since 2012, both human and mouse ENCODE projects have initiated related programs to broaden and deepen their respective efforts [59, 60]. Based on these, the ENCODE project has given researchers insight into how the human genome functions. As researchers learn more about the regulation of gene activity and how genes are expressed, the effects of the entire genome on human health will become clear. Recently, ENCODE has incorporated and processed the data from Roadmap Epigenomics Project which accord to ENCODE standards. ENCODE data are available at ‘encodeproject.org’ and can be visualized using the University of California, Santa Cruz (UCSC) genome browser (genome.ucsc.edu).

## Epigenome research projects: advances from ENCODE

### Roadmap epigenomics program

**Fig. 3:**
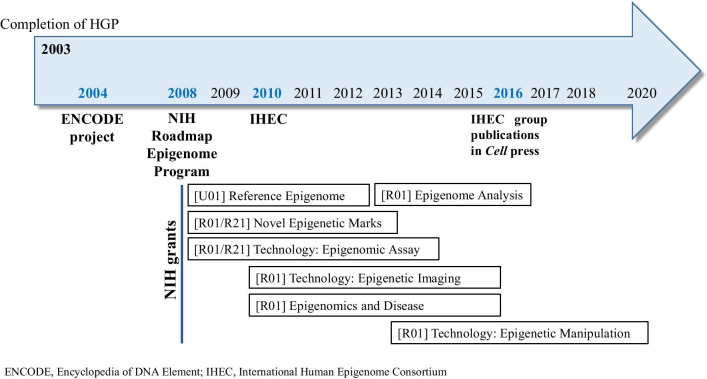
Perspectives of epigenome research. Epigenome researches in the past and in progress

The ENCODE project produced a number of papers in high-profile journals, e.g., Nature, Science, Cell, Genome Biology, Genome Research, and the Journal of Biological Chemistry, reporting over 1,650 experiments using 147 cell lines detailing functional features, including gene transcription, expression, transcription factor binding factors, chromatin conformation, DNA methylation, histone modification, and more [61, 62]. The large-scale mapping of epigenome started in 2003 as a part of ENCODE; many researchers have suggested the importance of studies of functional elements in the epigenome and associations with development and disease [63]. Epigenomics is the study of the complete set of epigenetic modifications at the whole genome level, known as the epigenome [64]. Improvements in research technology for genome-scale analyses have prompted researchers to develop a roadmap for gene regulation and other regulatory elements. Five years after launching ENCODE, the NIH funded a second large public project referred to as the ‘Roadmap Epigenomics Program (2008) (www.roadmapepigenomics.org)’ (Fig. 3), which has already contributed 61 complete epigenomes as of 2012, and the compilation of the “Human Epigenome Atlas” is in progress. Unfortunately, oral and maxillofacial regions have been excluded from the atlas work; therefore, more active genome-wide studies using appropriate dental samples and data processing techniques in the dental field are needed to keep pace with current advances. Roadmap Epigenome data sets are available at National Center for Biotechnology Information Gene Expression Omnibus (www.ncbi.nlm.nih.gov/geo/roadmap/epigenomics).

### International human epigenome consortium

Following the NIH Roadmap Epigenomics Program (2008), the International Human Epigenome Consortium (IHEC) was launched in 2010 (Fig. 3) [65]. IHEC is a global consortium, which aims to provide a free access to high-resolution reference human epigenome maps for a broad spectrum of human cell types and a wide range of developmental stages with respect to environmental variation. Several countries, including Canada, European Union, Germany, Hong Kong, Japan, Singapore, South Korea, and USA, have participated in contributing reference epigenomes. Recently, the IHEC coordinated the production of a catalog of their studies (http://www.cell.com/consortium/IHEC), which describes epigenetic molecular mechanisms and computational tools for various disease conditions. More details are available at IHEC official homepage (http://ihec-epigenomes.org/).

### Next-generation sequencing approaches to epigenomics

The epigenetic field was an early adopter of new molecular biological technology. Next-generation sequencing (NGS) techniques have placed epigenomics at the forefront of current research. NGS-based epigenome studies have provided detailed and multidisciplinary views of epigenetic modifications for the genomes of various cell types [66]. Representative epigenetic factors, i.e., DNA methylation and histone modifications, are routinely investigated genome-wide using NGS technology. Compared to the histone modification profile, relatively more approaches for DNA methylation profiling using NGS platforms have been introduced.

#### DNA methylation profiling

With the development of microarray hybridization technology, DNA methylation studies have been scaled up to the genome-wide level. It is possible to construct genomic maps of DNA methylation with a single base resolution [67]. Genome-wide analyses of DNA methylation are divided into three general types, i.e., affinity enrichment, methyl-sensitive restriction enzymatic reaction, and direct bisulfite conversion [68].

1. Affinity enrichment-based methods: This technique uses methyl CpG binding domain (MBD) proteins or 5′-methyl cytosine-specific antibodies to enrich methylated DNA regions.
  Microarray: MBD or Methylated DNA Immunoprecipitation (MeDIP) ChIP.
  High-throughput sequencing: MBDCap-seq [69], MethylCap-seq [70], MeDIP seq [71].
2. Restriction enzyme-based methods: This technique uses the differential digestion properties of methylation-sensitive restriction enzymes (MREs). We can search MREs in the New England BioLabs databases (https://www.neb.com/tools-and-resources/selection-charts/dam-dcm-and-cpg-methylation). MRE-based methods have been coupled with NGS techniques to estimate genome-wide DNA methylation levels [72].
3. Bisulfite conversion methods: This technique is based on changes in the methylated and unmethylated cytosine with sodium bisulfite treatment. It is used to study genome-wide methylation, e.g., by methylation arrays, whole-genome bisulfite sequencing (WGBS) [73], and reduced-representation bisulfite sequencing (RRBS) [74].

#### Histone modification profiling

By combining chromatin immunoprecipitation (ChIP) assays with sequencing technology, ChIP sequencing (ChIP-Seq) is a powerful tool for analyzing genome-wide DNA binding sites for transcription factors and other associated proteins [75]. ChIP-seq recognizes the binding sites of DNA- or histone-associated proteins and provides a global binding map for given proteins. Unlike other approaches, ChIP-seq does not require prior knowledge, e.g., to make probes from known sequences. The application of NGS to ChIP has provided insights into the development of disease and biological mechanisms on a genome-wide scale [76].

#### Non-coding RNAs (ncRNAs) profiling

Advances in sequencing technologies enabled to discover thousands of unannotated non-coding transcripts. RNA sequencing (RNA-seq) is a major transcriptome profiling system than polymerase chain reaction (PCR) or microarray, and useful to identify the novel transcript. For antisense ncRNA identification, strand-specific RNA-seq is recommended [77].

## Epigenetic studies in periodontology: from specific genes to a genome-wide approach

Epigenetics is regarded as a new frontier in dentistry [78]. In particular, the periodontal research field adopted epigenetic approaches relatively early. Epigenetic events regulate gene expression by chromatin remodeling and selective gene activation or inactivation [79]. These events may induce modifications in the cytokine profile and immune mechanisms and may thereby contribute to the pathogenesis of various infectious and inflammatory diseases [80–84]. Because most periodontal diseases are initiated by the bacterial infection and inflammatory processes, which are associated with individual differences and environmental risk factors, epigenetic studies could help clarify the pathogeneses [4, 85].

### DNA methylation and periodontal disease

Most studies of DNA methylation have focused on specific genes in human samples using methylation-specific PCR (MSP), bisulfite-specific PCR (BSP), methylation-sensitive restriction enzyme-PCR (MSRE-PCR), and combined bisulfite restriction analysis (COBRA) (Table 1). However, this approach was limited for analyses of the entire region at the low-throughput level. Advances in sequencing technology have broadened the target for epigenetic studies from specific genes to genome-wide analyses. Barros et al. performed a genome-wide CpG methylation assay to compare healthy gingival tissues and periodontally diseased gingival tissues [4]. De Souza et al. tried high-throughput DNA analysis in chronic periodontitis, indicating that variations in DNA methylation pattern between healthy and periodontitis are higher in genes related to the immune-inflammatory process [86]. In addition, as combined analysis about both DNA-methylation and gene-expression patterns, RNA and bisulfite sequencing were performed [39, 87]. In this study, comprehensive analysis including differential gene expression, DNA methylation, between-group correlation, gene set enrichment, and protein–protein interaction indicated that smoking-related changes in DNA methylation patterns and subsequent alterations in the extracellular matrix-related genes may be causally related to the increased susceptibility to periodontitis in smokers [39]. And in the comparison of periodontitis and peri-implantitis group, the analysis indicated that periodontitis and peri-implantitis share biological similarities; however, hierarchical clustering between both disease groups revealed distinct molecular characteristics. These differences could be deduced from structural differences in natural tooth-bone and implant-bone. Additionally, smoking differentially affected periodontitis and peri-implantitis for impaired host-defense mechanisms [87].

**Table 1:** Overview of epigenetic studies in periodontology

Specific gene			
Publication	Methods	Targets	Results
*(1) DNA methylation*			
Oliveira et al. [107]	Methylation Specific PCR (MSP)	Interleukin (IL)-8	Individuals with chronic periodontitis, independent of smoking habit, have a higher percentage of hypomethylation of the IL-8 gene
Andia et al. [108]	MSP	IL-8	A marked hypomethylated status in the promoter region of the IL-8 gene was found in aggressive periodontitis
Loo et al. [109]	MSP	E-cadherin, COX-2	The methylation of CpG islands in E-Cadherin and COX-2 genes in periodontitis patients occurs more frequently in periodontitis patients
Zhang et al. [110]	Pyrosequencing	(Interferon)IFN-γ	A hypomethylation profile within IFNG promoter region is related to an increase of IFNG transcription present in the chronic periodontitis
Zhang et al., 2010 [111]	Bisulfite-specific PCR (BSP)	Prostaglandin-endoperoxide synthase (PTGS)2	The increase of methylation in chronic periodontitis was associated with a level of PTGS2 mRNA expression
de Oliveria et al. [112]	Methylation-Sensitive Restriction Enzyme-PCR (MSRE-PCR)	Toll-like receptor (TLR)2, TLR4	No significant differences on DNA methylation status of the TRL2 and TRL4 gene promoters in periodontitis
Viana et al. [113]	MSP, BSP	IFN-γ, IL-10	No significant differences on DNA methylation status of the IFN-γ and IL-10 genes were observed
Ishida et al. [114]	BSP	IL-6	Hypomethylated status of a single CpG in the IL‐6 promoter region may lead to increased levels of serum IL‐6, implicating a role in the pathogenesis of chronic periodontitis
de Faria Amormino et al. [115]	MSRE-PCR	TLR2	Positive correlation between the TLR2 methylation frequency and probing depth was observed in periodontitis
Stefani et al. [116]	MSP	IL-6	The high expression of IL-6 is an important factor related to chronic periodontitis, but was not associated with methylation status
Zhang et al. [117]	BSP, Pyrosequencing	Tumor necrosis factor (TNF)-α	Increased methylation of TNF-α in chronic periodontitis compared to those with gingival health
Baptista et al. [88]	COmbined Bisulfite Restriction Analysis (COBRA)	suppressors of cytokine signaling (SOCS)-1, long interspersed nuclear element (LINE)-1	Different DNA methylation status of SOCS1 and LINE-1 was observed in infected oral environment
Andia et al. [89]	MSRE-PCR	SOCS-1,3 and LINE-1	DNA methylation levels for SOCS1 and SOCS3 did not differ between healthy and periodontitis groups
Kobayasi et al. [118]	BSP	IL-6	The increased expression of IL-6 gene may be related to IL-6 promoter hypomethylation in periodontitis
Kojima et al. [119]	BSP	TNF-α	The hypermethylated status of CpG motifs in the TNF-α gene promoter in blood cells may be unique to Japanese adults with chronic periodontitis
Schulz et al. [120]	PCR array	22 inflammatory candidate genes including CCL25 and IL17C	Differential methylation pattern for CCL25 and IL17C in periodontitis was observed
Asa'ad et al. [121]	Pyrosequencing	LINE-1, COX-2, IFN-γ, TNF-α	Periodontal therapy did not influence gene expression methylation of TNF‐α, IFN‐γ and LINE‐1 levels; however, it significantly reduced COX‐2 methylation levels
Shaddox [122]	Pyrosequencing	TLR signaling genes (FADD,MAP3K7,MYD88,IL6R,PPARA,IRAK1BP1,RIPK2)	Significant differences in methylation between LAP patients compared to healthy controls were observed
Li et al. [123]	Pyrosequencing	MMP-9 and TIMP-1	Positive correlation between methylation levels of MMP-9 CpG islands and the severity of chronic periodontitis was found

Genome-wide		
Publications	Methods	Results
Barros et al. [4]	CpG methylation array	The methylation status of some of these CpG regions suggests linkages with inflammatory pathways associated with acute and chronic inflammatory states
De Souza et al. [124]	Genome-wide methylation assay	Variations in DNA methylation between healthy and periodontitis cases are higher in genes related to the immune-inflammatory process
Cho et al. [39]	RNA sequencing, Reduced representation bisulfite sequencing (RRBS)	Smoking may change the transcription and methylation states of extracellular matrix (ECM) organization-related genes, which exacerbated the periodontal condition
Cho et al. [87]	RNA sequencing, RRBS	Periodontitis and peri-implantitis share biological similarities; however, hierarchical clustering between the two disease groups revealed distinct molecular characteristics

Publications	Methods	Target	Results
*(2) Histone modification*			
Cantley et al. [90]	Histone deacetylase inhibitor (HDACi) treatment to mouse model of periodontitis	Histone deacetylase	HDACi effectively suppresses bone loss in the mouse model of periodontitis
Larsson et al. [125]	Chromatin immunoprecipitation (ChIP) assay	IL-10	LPS stimulation of B cells resulted in an increase in acetylation of H4 and methylation of H3
Martins et al. [85]	Immunohistochemistry about histone modification in mouse periodontitis model	Acetylation in H3K9	Toll-like receptors 1, 2, and 4 and the nucleotide-binding oligomerization domain protein 1 induced histone acetylation in oral epithelial cells

Publications	Methods	Target	Results
*(3) Long non-coding RNAs (lncRNAs)*			
Gholami et al. [126]	Real-time PCR	ANRIL lncRNA	Expression of ANRIL was significantly lower in the blood of periodontitis
Li et al. [95]	Real time PCR	MALAT1 lncRNA	MALAT1 up-regulated in the gingival tissue of chronic periodontitis MALAT1 enhances inflammatory cytokine production via miR-20A and releasing TLR4
Wang et al. [94]	Real-time PCR	POIR lncRNA	POIR lncRNA may act as a competing endogenous RNA for miR-182, leading to derepression of target gene, FoxO1

### Histone modifications and periodontal disease

The majority of epigenetic studies in periodontology have focused the changed in DNA methylation of target genes [42, 88, 89]. Generally, overexpressed pro-inflammatory cytokine genes in the inflamed gingival tissue showed decreased DNA methylation level. Relatively few studies have focused on histone modifications and periodontal disease compared with DNA methylation (Table 1). Some studies have used an animal periodontitis model with a histone deacetylase inhibitor (HDACi), suggesting the potential to use HDACi for therapeutic purposes [90], and evaluated histone modifications in periodontitis with periodontopathic bacteria [85]. Owing to the general lack of studies of histone modifications, further genome-wide studies are needed to confirm the effects of histone modifications in the field of periodontal research.

### Non-coding RNAs and periodontal disease

Long non-coding RNAs (lncRNAs) refers to a large class of transcripts over 200 nucleotides not encoding proteins [91]. Several studies have reported the aberrant expression of lncRNAs including POIR, MALAT1, ANRIL, FGD5-AS1, NEAT1, and NKILA in periodontitis patients compared with healthy subject [92, 93]. POIR lncRNA in periodontitis modulates osteoblast differentiation via miR-182 and subsequent reactivation of target gene, FoxO1 [94]. MALAT1 lncRNA increased in chronic periodontitis modulating expression of pro-inflammatory cytokines via miR-20a and TLR pathway [95]. In addition, some studies have reported association between lncRNAs SNPs and inflammatory factors of periodontitis [96].

## Periodontal disease and NCDs from an epigenetic perspective

**Fig. 4:**
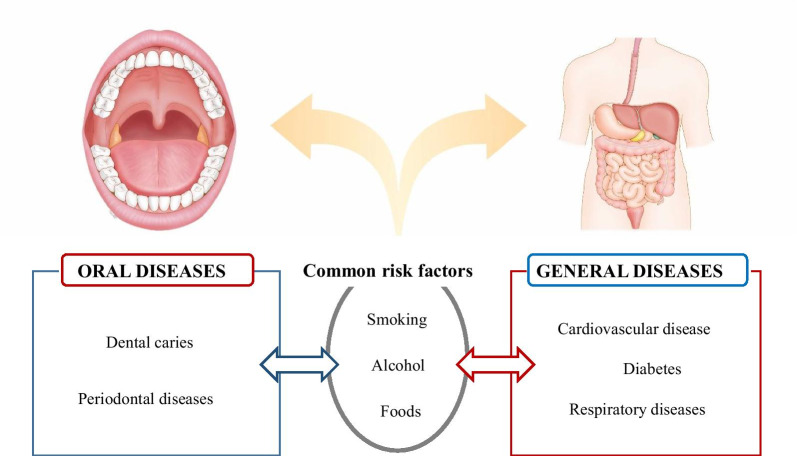
Common risk factors approach in the oral and general diseases. Periodontal disease and non-communicable diseases such as cardiovascular disease, diabetes, and respiratory disease share common risk factors including smoking, alcohol consumption, and poor dietary habits

Although periodontal disease is a major global public health issue with a high prevalence, high economic cost, and whole body consequences, it has a tendency to be neglected with respect to the total healthcare budget [97]. Non-communicable diseases (NCDs) are defined as medical conditions or diseases that are non-infectious or non-transmissible, which could refer to chronic diseases with long durations and slow progress [98]. Chronic NCDs are the world’s leading causes of death and disability. Representative NCDs are cardiovascular diseases, respiratory diseases, and diabetes (Fig. 4). Many NCDs can be prevented by reducing common risk factors such as smoking, alcohol use, unhealthy foods, and physical inactivity. Approaches for the control and prevention of NCDs have been outlined in the United Nations high-level meeting on NCDs in 2011, and governments subsequently adopted a goal of a 25% relative reduction in premature mortality from NCDs by 2025 (the “25 × 25” goal) [99]. To strengthen national efforts to reduce the load of NCDs, the 66th World Health Assembly developed the WHO Global Action Plan for the Prevention and Control of NCDs 2013–2020 [100], which is mostly concerned with the control of lifestyle factors. It is thought that lifestyle and environmental factors, beyond genetic traits, interact to determine the development of NCDs. As epigenetic changes are related to lifestyle and environmental factors, there may be a relationship between epigenetics and NCDs, and some recent human studies have reported that several NCDs have epigenome-wide associations with DNA methylation [101].

Most previous studies have focused on the inflammatory-immune background of oral diseases such as dental caries and periodontitis in response to the bacterial challenges. However, there has been evidence that oral disease may not be directly associated with oral microbiome [42]. Current evidence indicates that epigenetics plays an important role in the disease mechanism and clinical implication (Table 1). Interestingly, NCDs and oral diseases share common risk factors, such as smoking, alcohol, and poor diet (Fig. 4). The most common risk factors are environmental factors, which are candidate epigenetic factors [102]. Many reports have suggested that the common risk factors and shared pathological mechanisms could propose that there may be potential synergistic control of NCDs and periodontal disease [103–106]. We also found that smoking-related changes in DNA methylation patterns and subsequent alterations in the expression of genes are causally related to the increased susceptibility to periodontitis and peri-implantitis based on an NGS analysis [39, 87]. These results provide a rationale for conducting further studies using larger cohorts, the results of which will further elucidate the critical mechanisms underlying the observed patterns related to environmental hazards. In addition, future studies may explore prevention of the occurrence and progress of the disease. Likewise, epigenetic research implies innovative insights to find novel biomarkers for early diagnosis, treatment, and prognosis.

## Future perspectives

The ENCODE project provided researchers with new insight into how the genome functions, and allowed Epigenomic mapping using a systems biology approach to become an active international research trend. The field utilizes various new technologies to acquire information that cannot be revealed using classical forward or reverse genetic approaches. With the development of advanced sequencing technologies and bioinformatics applications, ‘omics’ profiling has catalyzed the concept of personalized medicine. Although international consortium is doing well now, it still has some limitations. As epigenetic landscape can be changed by environment factors, epigenome data has large individual variations that make it difficult to determine the reference. For accurate reference of epigenome, a more comprehensive analysis with well-classified and environmentally controlled cells or tissues is needed. In addition, complementary profiling of epigenome, gene expression, and phenotype on target disease are required for the integration of mapping activities. Genetic and environmental interactions in periodontology are well under way, and epigenetic factors are expected to have increased roles in the diagnosis and treatment of periodontal disease, thereby extending the potential for personalized medicine. In order to actualize the personalized medicine, it is necessary to comprehensively analyze multi-omics data set including genome, epigenome, transcriptome, metabolome, microbiome, and proteome, etc., and to collaborate harmoniously with bioinformaticians, scientists, and clinicians. Furthermore, advances in development of user-friendly and easy-to-handle software to utilize massive amount of data are also important for the practical application in various fields of research. Although epigenetic studies in periodontal disease have been conducted recently, in-depth integrated analysis with other information already obtained will create synergistic effects. In addition, targeted editing of epigenome to regulate gene expression could be the next possible step for disease control.

## Conclusion

The comprehensive understanding of periodontal disease benefited from ENCODE and related epigenome research projects. Although these advances have not yet been realized in clinical settings, many trials have been performed to identify biomarkers for diagnosis as well as epi-drugs for the treatment and prevention of periodontal disease. These findings will provide novel insight into the etiology and pathologic mechanism by genetics and epigenetics. Several epidemiologic evidences maintain that close link exists between NCDs and oral diseases. Furthermore, epigenetics, along with common risk factors, suggests that there may exist systemic and oral cross talk. In line with global research trends, further comprehensive studies of the functional and regulatory elements involved in periodontal health conditions and systemic diseases by joining with the ENCODE project will help to identify new biological mechanisms and thereby to develop diagnostic biomarkers and therapeutic strategies to promote oral health and general health, and expand the scope of ongoing large-scale research projects.

## Data Availability

Not applicable.

## Abbreviations

ENCODE: Encyclopedia of DNA Elements
HGP: Human Genome Project
IHEC: International Human Epigenome Consortium
NGS: Next-generation sequencing
ncRNAs: Non-coding RNAs
NCDs: Non-communicable diseases
